# Well-Being in Life and Well-Being at Work: Which Comes First? Evidence From a Longitudinal Study

**DOI:** 10.3389/fpubh.2020.00103

**Published:** 2020-04-09

**Authors:** Dorota Weziak-Bialowolska, Piotr Bialowolski, Pier Luigi Sacco, Tyler J. VanderWeele, Eileen McNeely

**Affiliations:** ^1^Sustainability and Health Initiative (SHINE), Department of Environmental Health, Harvard T. H. Chan School of Public Health, Boston, MA, United States; ^2^Human Flourishing Program, Harvard T. H. Chan School of Public Health, Institute for Quantitative Social Science, Harvard University, Cambridge, MA, United States; ^3^Department of Humanities, IULM University Milan, Milan, Italy; ^4^metaLAB (at) Harvard, Harvard University, Cambridge, MA, United States; ^5^Fondazione Bruno Kessler, Trento, Italy; ^6^Berkman-Klein Center for Internet & Society, Harvard University, Cambridge, MA, United States; ^7^Department of Epidemiology, Harvard T. H. Chan School of Public Health, Boston, MA, United States

**Keywords:** well-being in life, well-being at work, health, job and life satisfaction, happiness, meaning and purpose in life and at work, social relationships

## Abstract

Understanding reciprocal relationships between specific arenas in life and at work is critical for designing interventions to improve workplace health and safety. Most studies about the links between dimensions of well-being in life and at work have been cross-sectional and usually narrowly focused on one of the dimensions of the work-life well-being link. The issues of causality and feedback between life and work well-being have often not been addressed. We overcome these issues by measuring six aspects of well-being for both the work arena and life in general, using longitudinal data with a clear temporal sequence of cause and effect, and by explicitly accounting for feedback with potential effects in both directions. Nine hundred and fifty-four Mexican apparel factory workers at a major global brand participated in two waves of the Worker Well-Being Survey. Data on life satisfaction and job satisfaction, happiness and positive affect, meaning and purpose, health, and social relationships in life and at work were used. Lagged regression controlling for confounders and prior outcomes was employed. Sensitivity analysis was used to assess the robustness of the results to potential unmeasured confounding. For the relationships between life satisfaction and job satisfaction and between happiness in life and happiness at work effects in both directions were found. Nevertheless, indication of a larger effect of life satisfaction on job satisfaction than the reverse was obtained. For depression and meaning in life, there was evidence for an effect of life well-being on work-related well-being, but not for the reverse. For social relationships and purpose, there was evidence for an effect of work-related well-being on life well-being, but not the reverse. Relationships based on the longitudinal data were considerably weaker than their respective cross-sectional associations. This study contributes to our understanding of the nature of the relationship between aspects of well-being in the arenas of life and work. Findings from this study may facilitate the development of novel workplace programs promoting working conditions that enable lifelong flourishing in life and at work.

## Introduction

Although the influence of work on occupational health and safety has been long recognized (1), importance of work for well-being has been gaining scientific attention only recently (2–6). The impact of employee health on work has been traditionally examined through the lenses of physical and mental disabilities that limit chances for performing certain jobs (7–9). Recently the topic of worker well-being has been gaining attention in the field of occupational health. The United States Centers for Disease Control and Prevention's National Institute for Occupational Safety and Health (CDC/NIOSH) launched in 2011 the Total Worker Health® program that integrates protection against work-related and health hazards with promotion of injury avoidance and illness prevention to advance worker well-being (10–12). The World Health Organization introduced the Model for Action, which advocates for workers' health, safety and well-being on and off the job (13). Similar conceptual idea, highlighting the importance of achieving living and working conditions that enable people to engage and thrive at work over their lives, lies behind the concept of sustainable work over the life course which was introduced in the European Union to help people maintain health, develop skills and achieve financial security, work–life balance, meaningful work, and sense of self-fulfillment in the workplace (14). These worker well-being promoting initiatives emerge in labor market policies (14) and are subsequently integrated into companies' strategies (12, 15).

We argue that understanding the reciprocal relationships between well-being aspects at work and in life is critical to design policies to improve not only workplace health and safety but also employee satisfaction and well-being. Unfortunately, studies about the links between dimensions of well-being in life and at work have been usually narrowly focused on one of the dimensions of the work-life well-being link and additionally—have been mostly cross-sectional making causal inference implausible. The aim of this paper is to offer a more holistic outlook of the relationships between well-being at work and well-being in life by evaluating reciprocal relationships between six dimensions of well-being (such as life satisfaction, happiness, meaning, purpose, mental health, and social relationships) and their work-related counterparts while considering the bi-directional effects between work and life for each of the dimensions over time. This perspective contrasts with numerous well-being studies that not only limit well-being to a single life-related measure but also conceal the role of work as a driver for human flourishing and disregard the value of promoting flourishing in life to enhance flourishing at work. Consequently, in this article we hypothesize that for each of the six dimensions, significant reciprocal relationship between well-being in life and well-being at work can be established. In other words, we test a hypothesis that well-being while at work positively influences well-being in life and well-being in life is beneficial for well-being while at work.

## Literature Review

One of the most examined relationships between well-being and work has been the one between subjective well-being (SWB)^1^ and job satisfaction (16–19). This relationship has been subject to scrutiny over the past decades, with early contributions dating back to the 1950's (20). Previous studies, however, identified only a modest to moderate association between SWB and job satisfaction (16, 19). Limited evidence, however, is available for the relationship between other dimensions of well-being in life and their counterparts related to well-being at work.

Many existing studies are also hardly conclusive due to serious methodological limitations. Most of the studies have been based on cross-sectional study designs (18, 21), which rendered it impossible to establish any causal link. Limited longitudinal research carried out so far mainly focused on the relationship between the broad concepts of life satisfaction and job satisfaction without delving into its constituents [see (16) for a review] or work-life conflict (22, 23), avoiding a scrutinized study of other aspects of well-being in the arena of life and work.

Theoretical and empirical lack of agreement on the directionality of the relationship between well-being and work further complicates the interpretation and assessment of the findings (16). For instance, the part-whole theory (24, 25) posits that specific aspects of life (e.g., work) influence well-being, whereas the dispositional approach (26, 27) claims that it is well-being that has a causal effect on specific aspects of life (e.g., work).

Additionally, regarding the directionality of the relationship, a heated dispute arose between proponents of the spillover approach, advocating for a reciprocal, positive relationship between specific aspects of life (e.g., work) and well-being (16, 28), the compensation approach, assuming that dissatisfaction in one sphere is compensated by search for enrichment in the other (thus envisaging a negative relationship), and the segmentalist approach, making a case for a lack of relationships between the two areas (29). Current evidence is thus inconclusive and thus all hypotheses about the cause and the effect remain plausible.

The conceptual and operational definitions of well-being in life and well-being at work have been refined more recently as well. The definitions shifted from early characterizations in broad affective terms to more articulate, conceptually sharper ones (30), which provide relatively robust and consistent frameworks necessary for a scientific analysis (16). For example, consideration of job and life satisfaction is now combined in notions of employee well-being (31–33) and more well-being interventions are proposed to ensure that workers are both happy (or high in well-being) and productive (have high performance) (34, 35).

However, the nature of the work-life link is still unclear. Despite strong evidence provided by Bowling et al. (16) that the effects are bi-directional and life satisfaction affects job satisfaction more than job satisfaction affects life satisfaction, the issue of the direction of causality and strength of bi-directional relations between dimensions of well-being at work and well-being in life remains fundamentally open and unexplored. Although recent research has extended our contextual knowledge about the possible effects on the job-life satisfaction relationship [for example the effects of: burnout (36, 37), positive affect or negative affect (19, 28), job importance (38), work-family conflict (19, 28), work-life balance (39, 40), workplace friendship (41, 42), job insecurity (43), and even geographical remoteness (44)], a more comprehensive approach—as advocated also by Neve et al. (2) is needed. However, it is worth noting that a distinction between workplace well-being from general well-being has been recently recognized (33). Still, limited evidence on how particular aspects of general well-being affect their counterparts while at work and vice versa is available.

Consequently, this paper offers the following contributions in this relatively under-explored direction. First, by carrying out a longitudinal analysis it provides more robust evidence on the causal relationships between job and life satisfaction. Second, by studying in depth other aspects of well-being in the work and life sphere, such as happiness (45), meaning (46), purpose (47), mental health (48) and social relationships (49), our results provide an innovative framework for the analysis of the job vs. life dimensions of well-being studied in the literature as well as evidence for their causal directionality.

## Materials and Methods

### Data Source and Sample Size

The analysis builds on the first two waves of the Worker Well-Being Survey (WWBS), a tool designed to track workers' well-being, administered in the Levi Strauss & Co.'s supplier in Mexico. The first wave of the WWBS was administered in February 2017, and the second one in March 2018.

Workers completed surveys in a private space inside the factory on tablets either connected directly to secure servers via the internet or using an offline app. In this way, all information was kept confidential. During survey administration, groups of workers were released from their line positions (e.g., one production line at a time) to come to the survey stations. A communication campaign took place prior to survey activities to invite workers to participate in the survey. The results were reported in aggregate to workers and the factory after tabulation and analysis. The workers' decision to participate in the survey was voluntary and was not disclosed to management. All workers signed an informed consent. The study was approved by Harvard T.H. Chan School of Public Health Institutional Review Board.

Nine hundred fifty-four apparel workers participated in both waves of the WWBS. Descriptive statistics of the sample are presented in Table 1. Data are available from the corresponding author upon request.

**Table 1:** Descriptive characteristics of the sample.

**Characteristic**	**Statistic**
Gender (female)	53.7%
Age	
Below 25	21.4%
25–34	28.6%
35–44	32.8%
45+	27.3%
Marital status (married)	45.7%
Education (at least high school)	31.8%
Having children under the age 18 currently living in the household	67.9%
Being a primary caretake for a parent or an elderly currently living in the household	47.6%
Job Tenure	
Up to 1 year	25.7%
From 1 up to 3 years	28.3%
From 3 up to 5 years	13.0%
More than 5 years	33.0%

### Measures

In analyzing the relationships between life-related well-being factors and their job-related counterparts, we distinguished six aspects of well-being: (1) life satisfaction and job satisfaction, (2) happiness, (3) meaning, (4) purpose, (5) social relationships, and (6) mental health. Questions measuring the first five aspects originated from the flourishing index (50–52), while the question measuring health was adopted from the set of healthy days questions of the Health-Related Quality of Life instrument (53). For each well-being question a work-related counterpart was used. Specifically, questions from an adapted version of the Positive and Negative Affect Scale (PANAS) (54) referring to work domain, i.e., from the Job-Related Affective Well-Being Scale (55) were used, as well as a question about job satisfaction and meaning and purpose at work (56, 57).

The full set of questions used to measure specific dimensions of well-being from both a life- and job-related perspective is presented in Table 2. Table 3 presents descriptive statistics of the variables in the study. Correlation matrix of the measures is provided in Table A1 in the Appendix.

**Table 2:** Job-related and out-of-job variables measuring well-being in life and well-being at work.

**Dimension of well-being**	**Job-related variable**	**Out-of-job variable**
Life satisfaction	Job satisfaction: all in all, how satisfied would you say you are with your job? (0 = Not Satisfied At All, 10 = Completely Satisfied) (56)	Life satisfaction: overall, how satisfied are you with life as a whole these days? (0 = Not Satisfied at All, 10 = Completely Satisfied) (50)
Happiness	Happiness at work: at work yesterday, or the last day I worked, I felt happy; dichotomized: 1 = frequently or all the time, 0 = not at all or occasionally (54, 55)	Happiness in life: in general, how happy or unhappy do you usually feel? (0 = Extremely Unhappy, 10 = Extremely Happy) (50)
Meaning	Meaningful job: my job is meaningful; originally measured on a Likert scale; in the analysis dichotomized: 1 = agree, 0 = disagree (56, 57)	Meaning in life: overall, to what extent do you feel the things you do in your life are worthwhile? (0 = Not at All Worthwhile, 10 = Completely Worthwhile) (50)
Purpose	Feeling purposeful at work: at work yesterday, or the last day I worked, I felt that my job is purposeful; dichotomized: 1 = frequently or all the time, 0 = not at all or occasionally (56, 57)	Purpose in life: i understand my purpose in life. (0 = Strongly Disagree, 10 = Strongly Agree) (50)
Close social relationships	Friends at work: at work yesterday, or the last day I worked, I felt close to other people; dichotomized: 1 = frequently or all the time, 0 = not at all or occasionally	Friends in life: i am content with my friendships and relationships (0 = Strongly Disagree, 10 = Strongly Agree) (50)
Mental health	Depressed at work: at work yesterday, or the last day I worked, I felt depressed; dichotomized: 1 = frequently or all the time, 0 = not at all or occasionally (54, 55)	Depressed in life: During the past 30 days, for about how many days did you feel sad or depressed? (dichotomized: 0 = none; 1 = at least 1 day) (53)

**Table 3:** Descriptive statistics of the variables in the study.

**Variable**	***T* = 1**	***T* = 2**
**Job related**		
Job satisfaction (0–10)	8.44 (2.41)	8.61 (1.99)
Happiness at work (% of yes)	68.0%	71.8%
Meaningful job (% of yes)	92.1%	90.1%
Feeling purposeful at work (% of yes)	72.5%	75.1%
Friends at work (% of yes)	70.4%	77.7%
Depressed at work (% of yes)	6.5%	9.9%
**Out-of-Job**		
Life satisfaction (0–10)	8.12 (2.84)	8.51 (2.14)
Happiness in life (0–10)	8.69 (2.24)	8.76 (1.94)
Meaning in life (0–10)	9.04 (1.94)	9.24 (1.64)
Purpose in life (0–10)	9.36 (1.86)	9.29 (1.72)
Friends in life (0–10)	9.09 (1.86)	8.97 (1.82)
Depressed in life (% of at least 1 day in a month)	48.0%	54.0%

*Means and standard deviations are reported for variables measured on 0–10 response scale*.

### Control Variables

It has been empirically shown that relationship between well-being and job attitudes may be different depending on gender, age, and education (19, 58–65) and together with job tenure, these variables are among the most commonly used as control variables in the organizational research (59). There is also evidence that marital status or having a family in general, especially in combination with a necessity of raising a child, is a discriminatory factor for happiness (66) and job attitudes (67–70). Similarly, caregiving to an elderly has a detrimental effect on well-being (71, 72), health (73), and job satisfaction and other job attitudes (74). Additionally, there are theoretical foundations and supporting empirical evidence that job demand and job control correlate with mental health and job attitudes (75, 76).

Consequently, in the analysis, we controlled for: (1) demographic variables: gender, age, marital status, education, having children below 18 at home, taking care of an elderly; and (2) job characteristics: job tenure, job demand (“I have too much work to do, to do everything well;” yes/no), job control (“I have a lot of say about what happens on my job;” yes/no), and work shift (day vs. otherwise).

In the longitudinal analysis, these variables were controlled at baseline in Wave 1, in order to ensure that they were confounders and not mediators. In the cross-sectional analysis, they were measured simultaneously with the exposure and outcome so as to compare results with the more rigorous longitudinal analyses.

### Statistical Analysis

As the goal was to investigate a causal link between well-being in life and job-related well-being (i.e., how well-being in life influences job-related well-being and vice versa), longitudinal data was used and statistical approaches for modeling longitudinal data were employed. Contrary to analyses conducted on cross-sectional data, this approach offered more reliable causal evidence by virtue of the logical temporal sequence of cause and effect. However, as most of the empirical evidence in the field is based on cross-sectional data, we also ran secondary analysis on such kind of data, with the aim of assessing the level to which the relationship is inflated by the use of cross-sectional data.

The relationship was modeled using either linear regression model (for continuous outcomes), or logistic regression model (for dichotomous outcomes). With respect to dichotomous outcomes, odds ratios were reported; with respect to continuous outcomes, standardized regression estimates were provided to report standardized effect sizes.

The relationship between work-related well-being factors and their out-of-work well-being counterparts for continuous outcomes was modeled as follows:

(1) $$\begin{array}{r} WBW_{i,k}\left(T=2\right)={\alpha_{0}+{\alpha_{1}WBL_{i,k}\left(T=1\right)+\alpha }_{2}X}_{i}\left(T=1\right)+\eta_{i,k} \end{array}$$

(2) $$\begin{array}{r} WBL_{i,k}\left(T=2\right)={{{\beta_{0}+\beta }_{1}WBW_{i,k}\left(T=1\right)+\beta }_{2}X}_{i}\left(T=1\right)+\varepsilon_{i,k} \end{array}$$

and for dichotomous outcomes as follows:

(3) $$\begin{array}{r} prob\left[WBW_{i,k}\left(T=2\right)=1\right]=\\ \frac{1}{1+e^{-\left({\alpha_{0}+{\alpha_{1}WBL_{i,k}\left(T=1\right)+\alpha }_{2}X}_{i}\left(T=1\right)+\eta_{i,k}\right)}} \end{array}$$

(4) $$\begin{array}{r} prob\left[WBL_{i,k}\left(T=2\right)=1\right]=\\ \frac{1}{1+e^{-\left({\beta_{0}+{\beta_{1}WBW_{i,k}\left(T=1\right)+\beta }_{2}X}_{i}\left(T=1\right)+\varepsilon_{i,k}\right)}}, \end{array}$$

where *i* = 1,…, *N, k* = *1,…,6*.

Subscript *i* represents an individual, the variable *WBW* indicates one out of six (*k* = 1,…,6) work-related well-being factors, *WBL* is one out of six well-being in life factors. *X* is a vector of control variables including the first wave (*T* = 1) outcomes. α_1_reflects effects of an out-of-work well-being factor on a well-being at work outcome and β_1_ shows the effects of a well-being at work factor on a well-being in life outcome. α_2_ shows the association between control variables and the well-being at work outcome, β_2_ shows the association between control variables and a well-being in life outcome. η_*i*_ and ε_*i*_ are disturbance terms.

Robustness of the results was ensured by performing the sensitivity analysis (77) and through the design of the study's procedure to account for the common method bias (78). Sensitivity analysis was applied to assess the extent to which an unmeasured confounder would need to be associated with both the exposure and the outcome to explain away the observed association (77, 79). To this end, the *E*-value, which is a continuous measure of how robust the association is to potential uncontrolled confounders, was applied. The *E*-value is the minimum strength of association on the risk ratio scale that an unmeasured confounder would need to have with both the outcome and the primary exposure or independent variable, above and beyond the measured covariates, in order to explain away the observed association (77).

Regarding the common method bias, we accounted for it through the design of the study procedure (78). Specifically, although it was not feasible to account for a common rater and a common measurement context (as it was of crucial importance to get data from the same persons being in the same measurement context), we proximally and methodologically separated predictor, and outcome variables. Specifically, these variables were located in different sections of the questionnaire and different response scales were used, e.g., 4-point Likert scales, number of days, intensity scales, 0–10 Likert type scales (see Table 2), with different scale endpoints, and different verbal labeling. Additionally, the research team strived to ensure anonymity of respondents and reduce evaluation apprehension by (i) providing the choice to participate in the study and (ii) ensuring that participation would affect neither the employment conditions nor the employment status. Moreover, (iii) respondent might choose to not respond to any question(s) and (iv) withdraw without penalty at any time. Appropriate information about (i–iv) was conveyed in the communication campaign and also added to the invitation letter. Finally, the follow-up visits to the factories were conducted 1, 3 and 6 months after the survey administration and the individual interviews with selected workers were conducted to make sure that the workforce was not negatively affected by the participation in the study.

Analyses were performed using Stata 15.

## Results

The strength of the relationships based on the longitudinal data, controlling for prior outcome (Table 4), was found to be in each case weaker—and in the case of purpose and close social relationships also insignificant—than the strength of associations revealed from the cross-sectional analysis (Table 5). This suggests that evaluations based solely on cross-sectional data could over-estimate the actual strength of the relationships, which is consistent with previous research about job satisfaction and subjective well-being (16).

**Table 4:** Effect sizes (standardized estimates [std. est.] and odds ratios [OR]) and 95% confidence intervals (in parentheses) for the relationships between job-related well-being factors and their out-of-job counterparts—longitudinal results.

**Out-of-job factor (*T* = 1)**	**Job-related outcome (*T* = 2)**	**Job-related factor (*T* = 1)**	**Out-of-job outcome (*T* = 2)**
	Job satisfaction (std. est.)		Life satisfaction (std. est.)
Life satisfaction	0.142^***^ (0.069; 0.216)	Job satisfaction	0.088^*^ (0.011; 0.164)
	Happy at work (OR)		Happiness in life (std. est.)
Happiness in life	1.373^***^ (1.141; 1.653)	Happy at work	0.316^***^ (0.159; 0.473)
	Depressed at work (OR)		Depressed in life (OR)
Depressed in life	2.612^**^ (1.427; 4.782)	Depressed at work	0.892 (0.428; 1.860)
	Meaningful job (OR)		Meaning in life (std. est.)
Meaning in life	1.443^***^ (1.177; 1.769)	Meaningful job	0.235 (−0.051; 0.522)
	Feeling purposeful at work (OR)		Purpose in life (std. est.)
Purpose in life	1.085 (0.879; 1.339)	Feeling purposeful at work	0.220^**^ (0.056; 0.384)
	Friends at work (OR)		Friends in life (std. est.)
Friends in life	1.090 (0.893; 1.329)	Friends at work	0.168^*^ (0.012; 0.324)

*Each regression was controlled for: job control, job demand, gender, age, education, marital status, number of children, taking care of an elderly, job tenure, and work shift*.

* *p < 0.05*,

** *p < 0.01*,

*** *p < 0.001*.

**Table 5:** Effect sizes (standardized estimates [std. est.] and odds ratios [OR]) and 95% confidence intervals (in parentheses) for the association between job-related factors and their out-of-job counterparts—cross-sectional results.

**Out-of-job factor**	**Job-related outcome (cross-sectional)**		**Job-related factor**	**Out-of-job outcome (cross-sectional)**	
	***T*** **=** **1**	***T*** **=** **2**		***T*** **=** **1**	***T*** **=** **2**
	Job satisfaction (std. est.)			Life satisfaction (std. est.)	
Life satisfaction	0.292^***^ (0.226; 0.357)	0.291^***^ (0.221; 0.360)	Job satisfaction	0.322^***^ (0.250; 0.395)	0.325^***^ (0.247; 0.402)
	Happy at work (OR)			Happiness in life (std. est.)	
Happiness in life	1.478^***^ (1.139; 1.765)	2.084^***^ (1.696; 2.560)	Happy at work	0.339^***^ (0.195; 2.063)	0.731^***^ (0.564; 0.896)
	Depressed at work (OR)			Depressed in life (OR)	
Depressed in life	3.776^***^ (1.682; 8.477)	5.730^***^ (2.660; 12.347)	Depressed at work	3.718^***^ (3.266)	5.690^***^ (4.203)
	Meaningful job (OR)			Meaning in life (std. est.)	
Meaning in life	1.475^***^ (1.169; 1.861)	1.512^***^ (1.189; 1.924)	Meaningful job	0.626^***^ (0.359; 0.893)	0.442^***^ (0.186; 0.698)
	Feeling purposeful at work (OR)			Purpose in life (std. est.)	
Purpose in life	1.364^***^ (1.143; 1.627)	1.290^*^ (1.037; 31.604)	Feeling purposeful at work	0.286^***^ (0.135; 0.437)	0.185^*^ (0.030; 0.340)
	Friends at work (OR)			Friends in life (std. est.)	
Friends in life	1.643^***^ (1.357; 1.991)	1.304^**^ (1.077; 1.578)	Friends at work	0.437^***^ (0.287; 0.588)	0.267^**^ (0.088; 0.448)

*Each regression was controlled for: job control, job demand, gender, age, education, marital status, number of children, taking care of an elderly, job tenure, and work shift*.

* *p < 0.05*,

** *p < 0.01*,

*** *p < 0.001*.

The effect size of the influence of life satisfaction on job satisfaction was found to be higher (0.14) than the effect size of the influence of job satisfaction on life satisfaction (0.09). Happiness in life was found to influence feelings of happiness at work and it was also the case that feelings of happiness at work influence happiness in life (effect sizes could not be directly compared as the former was assessed with an odds ratio scale and the latter with a standardized difference scale). Therefore, for both relationships—life vs. job satisfaction and happiness in life vs. at work—there is evidence that the causal relations are bi-directional, despite having different strength in the two directions. Additionally, in terms of absolute strengths, causal links in the happiness sphere turn out to be considerably stronger than those in the satisfaction sphere.

For the remaining variables, however, the evidence suggests that the causal relations may be unidirectional, with the actual links emerging from the life to the job sphere or the other way around, depending on the specific dimension. Depression was shown to increase the probability of feeling depressed at work, but reports of feeling depressed at work were not found to increase probability of feeling depressed in general. Similarly, meaning in life was found to have an impact on meaning in job, but the reverse relationship was not found to be significant. Conversely, feeling purposeful at work was found to increase purpose in life but not the other way around. We also found evidence that feeling close to people at work contributes to a sense of improved social connections in life; however, the reverse relationship was not supported by our results. We provide some further exploration of the potential reasons for these uni-directional associations in the discussion.

### Sensitivity Analysis for Unmeasured Confounding

The E-values calculated for the longitudinal results (Table 6) indicate that most of the estimated associations were relatively robust to unmeasured confounding, which provides some further evidence of causality for those outcomes. The influence of job satisfaction on life satisfaction, and the relationship in the opposite direction, were moderately robust to potential unmeasured confounding. Only an unmeasured confounder that would be associated with both job satisfaction and life satisfaction by a risk ratio of 1.383 (the effect of job satisfaction on life satisfaction) and 1.536 (the effect of life satisfaction on job satisfaction), above and beyond the measured confounders, could explain away the observed association between life satisfaction and job satisfaction; weaker confounding could not. Confounders associated with both the outcome and exposure by risk ratios of 1.5-fold to 2-fold each would be required to explain the relationship between life and job: happiness, purpose, meaning, and friends, also pointing to relatively strong evidence of robustness to confounding for the link between life and job-related outcomes. An even stronger confounder would be necessary to explain away the relationship between depression in life and depression at work. The strength of association of this hypothetical confounder would have to reach at least 4.664 in terms of risk ratios, with both depression at work and depression in life in the model; and even to reduce the 95% confidence interval to include the null would require an unmeasured confounder association with both depression in life and depression at work by risk ratios of 2.2-fold each having already adjusted for all measured confounders.

**Table 6:** *E*-values for significant longitudinal effect measures and for corresponding CI limits.

**Out-of-job factor**	***E*-value for effect estimate**	***E*-value for CI limit**	**Job-related factor**	***E*-value for effect estimate**	***E*-value for CI limit**
	**Job-related outcome**			**Out-of-job outcome**	
	Job satisfaction			Life satisfaction	
Life satisfaction	1.536	1.328	Job satisfaction	1.383	1.115
	Happy at work			Happiness in life	
Happiness in life	1.621	1.338	Happy at work	2.000	1.582
	Depressed at work			Depressed in life	
Depressed in life	4.664	2.207	Depressed at work	—	—
	Meaning job			Meaning in life	
Meaning in life	1.693	1.389	Meaning job	—	—
	Feeling purposeful at work			Purpose in life	
Purpose in life	—	—	Feeling purposeful at work	1.743	1.29
	Friends at work			Friends in life	
Friends in life	—	—	Friends at work	1.604	1.12

*E-values are reported only for significant estimates. E-values indicate the strength of unmeasured confounding that would be necessary to invalidate the observed relationship and thus are not of interest when the measured effect is not significant*.

## Discussion and Conclusions

The results contribute to our understanding of the nature of the relationship between job satisfaction and life satisfaction, as well as between other dimensions of well-being in life and well-being at work. Generally, job satisfaction and happiness, but also purpose, and social connections while at work were found to influence their out-of-job counterparts 1 year later. With regard to the reverse direction, life satisfaction and happiness, but also depression and meaning in life were found to influence the work-related counterparts 1 year later. Thus, only for life satisfaction and happiness was there an evidence for effects running in both directions, confirming our research hypothesis about the reciprocal benefits between well-being in life and well-being at work. Other relationships were more unidirectional but not always necessarily indicative of an impact of work on life—the directionality more often acknowledged in the literature.

Our results are in some ways intuitive, but nonetheless they call for further scrutiny. Regarding happiness and life satisfaction, causal links in the happiness sphere in absolute terms turn out to be significantly stronger than those in the satisfaction sphere. This may be due to the fact that happiness, as a construct, also includes elements of coping resources and positive emotions (80), potentially eliciting more immediate connections between the work and life spheres. However, the feedback loops we found are in line with the two competing theoretical models of well-being: the bottom-up (situational) model and top-down (dispositional) model (27). The bottom-up model of well-being assumes that well-being is a sum of small pleasures. This implies, in turn, that life satisfaction and happiness may be situational and thus influenced by job satisfaction and positive affect while at work, respectively. Instead, according to the top-down model, each person tends to experience things in a particular, positive or negative way, thus well-being is dispositional (81). This is reflected in the way in which all life experiences are perceived, and in particular this implies that well-being is projected onto other variables. Specifically, the impact of life satisfaction on job satisfaction and of happiness in life on happiness while at work are anticipated. Consequently, life satisfaction and happiness may be both the cause (as in the top-down model) and the effect (as in the bottom-up model) of job satisfaction and positive affect while at work, respectively. This conclusion has been already made by other scholars, based on empirical evidence (82, 83) and on theoretical considerations conceptualized as the spillover model of well-being (18, 84, 85).

For depression, it was shown that depression in life increases the probability of feeling depressed at work, but reports of feeling depressed at work were not found to increase probability of feeling depressed in general. Depression in life is likely to manifest itself at work as its symptoms are neither temporarily limited to the periods spent out-of-work nor spatially confined to the non-working environment. However, depression at work may depend on very context-specific conditions that do not necessarily reflect a more general susceptibility to depression. In particular, the evidence on the effects of workplace stressors [e.g., prolonged job strain (86, 87), increased job demand (88, 89) and limited job control (90, 91)] on the development of depression is moderate but the level of exposure to stressors that seems to be generally needed to cause depression still requires further investigation (86, 92, 93).

Meaning in life was found to have an impact on meaning in one's job, but the reverse relationship was not corroborated. Thus, no support was found for the assertion by Steger and Dik (94), Duffy and Sedlacek (95), and Allan et al. (46) that meaning at work translates into greater meaning in life. Instead, our findings were in line with the top-down theory of subjective well-being (27) or the dispositional approach (26, 27), according to which global well-being translates into domain-specific well-being.

Specifically, meaning refers to overall relatedness in a larger sense, such as coherence and significance of one's experiences, whereas, purpose mainly refers to pursuit and aspiration of certain ends (57, 96). The one-directional causal links that we found seem to conform to intuition—with meaning, the more existential dimension, being driven by the life sphere, whereas purpose, the more goal-oriented dimension, being driven by the work sphere. This result appears to be in line with the findings of Steger and Dik (94), who report that both experiencing a calling and seeking life meaning are predictors of life meaning.

Similar to other studies (41, 42), we also found evidence that feeling close to people at work contributes to improved social connection in life. This finding corroborates Rumens' [(97), p. 1149] assertion that “workplace friendships contribute to human flourishing.” However, the reverse relationship was not supported by our results. This is again a result that conforms to intuition, as social connection at work will contribute to one's overall social well-being, but relationships outside of the workplace do not necessarily make workplace friendships any more likely. Additionally, social connection in the workplace may call for a more demanding social adaptation compared to the life sphere since, in the work environment, people have less control over the choice to associate with certain people or not, compared to their own out-of-work social environment, and the emotional control tasks in the former case are consequently more demanding (98). Additionally, it is natural that social relationships from work can spread (spill-over) into the life domain, while relationships from life are confined in the life domain. Despite recognition and effectiveness of word-of-mouth as a recruitment source (99, 100), one cannot expect to be able to often influence the hiring decisions of one's employer based on non-work-related friendship.

In contrast to the majority of other studies, we used longitudinal data thus making a substantial adjustment for confounding and control for work and life characteristics, which are known to correlate with aspects of both well-being at work and well-being in life. Although cross-sectional analyses [both ours and those of other authors; see e.g., (19)] suggest presence of moderate to strong bidirectional relationships, our longitudinal results provide evidence for potential effects in both directions, with effect sizes of roughly equal magnitude only for the relationships of life satisfaction-job satisfaction, and happiness at work-happiness in general/life. This confirms the findings of the meta-analysis of the relationship between job and life satisfaction conducted by Bowling et al. (16) on 11 (eight published and three unpublished) longitudinal studies, which may be more valid as they account for the logical and temporal sequence of cause and effect and for prior levels of outcomes. Moreover, our results here also suggest that only unidirectional effects exist concerning meaning, purpose, mental health, and social connectedness. Although the Worker Well-Being Survey was designed to target working adults and examine worker well-being, it must be also noted that our sample of Mexican manufacturing workers may reflect specific social conditions and cultural inclinations. Different samples, covering jobs with different characteristics and professional profiles, or taken in different geographical and socio-cultural contexts, might yield different results. The literature shows that cross-country variation in the dimensions, which are the object of this study, should be expected, with a possibly prominent role played by the local level of social capital (101–103). Likewise, work-related stress varies significantly across occupations (104, 105), and therefore—although we controlled for job demand and job control, which are well-known correlates of work-related stress and burnout (75, 76)—one can expect this source of variation to affect the relationship between well-being at work and in life. Consequently, there should be caution as to the generalizability of our results, and more research for different job profiles and in different geographical contexts should be carried out to gain a deeper insight. To this end, in particular, relatively more research effort should be directed toward longitudinal rather than cross-sectional studies, in order to improve our understanding of the structure of the causal relations between the work and life spheres of the other related variables of interest.

Our study made use of observational data. Most of the results presented in this study proved to be relatively robust to potential unmeasured confounding beyond a considerable number of measured potential confounders already included in the analyses. Thus, the evidence for causality was further strengthened. However, the results may still be subject to unmeasured confounding by personality, core self-evaluations, such as self-esteem, self-efficacy, locus of control and emotional stability (106, 107), as well as goal self-concordance (108). However, our sensitivity analysis indicates that, for an unmeasured confounder to explain the effect of the observed associations, it would have to be associated with both job-related and out-of-job well-being factors by a risk ratio equal in magnitude to at least 1.383, while in order to explain away the relationship between general depression and work-related depression an unmeasured confounder related to both measures of depression by more than four on the risk-ratio scale would be required.

We used two waves of data, which let us control for the baseline outcomes. However, future research should consider replicating the results using more waves of data to control also for the baseline exposure. Such analysis will provide further evidence for the robustness of our results.

Finally, in the analyses we relied on single item measures of well-being dimensions. Although it is a common practice to use multi-item measures in such a case, we argue that long instruments—despite the advantages of conceptual richness—are inferior to short instruments in studies focusing on a vast array of topics. Workers' well-being study measures well-being along with physical and psycho-social working conditions, work safety and occupational health, job burden, job autonomy, job resources, work-family conflict, and others. In such a setting, a less time-consuming instrument may be beneficial. By being short enough for practical use in the workplace, it facilitates company's efforts to improve the worker well-being (32). Criticism of short instruments—especially those with one item per domain—relates to elevated Type 1 and Type 2 error probabilities [see (109)] for evidence in the personality studies). Yet, such instruments can still be found in psychology (110, 111), educational psychology (112) and organizational behavior (113), among others. In the well-being field, it is worth noting that the United Kingdom Office for National Statistics—to avoid excessive costs and to enable widespread use—since 2011 includes a set of only four well-being questions in the UK National Survey (114).

In sum, we concede that work is just one arena to enhance well-being, however, given the amount of time spent at work across our lifetimes, seemingly a powerful one. Therefore, understanding the well-being ecosystem for impact areas and reciprocal relationships in life and at work is important to finding ways to intervene. Without this holistic view, the leverage points for optimizing well-being may be invisible or inadequate by an overemphasis or attribution to one sphere of influence only.

## Data Availability Statement

The datasets generated for this study are available on request to the corresponding author.

## Ethics Statement

The studies involving human participants were reviewed and approved by Harvard T. H. Chan School of Public Health Institutional Review Board. The patients/participants provided their written informed consent to participate in this study.

## Author Contributions

DW-B developed the study concept, contributed to data analysis and interpretation of the result, drafted the manuscript and approved the final version of the manuscript. PB contributed to data analysis and interpretation of the result, drafted the manuscript and approved the final version of the manuscript. PS contributed to interpretation of the results, drafted the manuscript and approved the final version of the manuscript. TV contributed to interpretation of the results, revised the manuscript and approved the final version of the manuscript. EM developed the study design, revised the manuscript and approved the final version of the manuscript.

### Conflict of Interest

The authors declare that the research was conducted in the absence of any commercial or financial relationships that could be construed as a potential conflict of interest.

## Figures and Tables

**Table A1:** Zero-order correlation matrix of the variables in the study (at *T* = 1).

**Variable**	**(1)**	**(2)**	**(3)**	**(4)**	**(5)**	**(6)**	**(7)**	**(8)**	**(9)**	**(10)**	**(11)**
Job satisfaction (1)											
Happiness at work (2)	0.273^***^										
Meaningful job (3)	0.222^***^	0.198^***^									
Feeling purposeful at work (4)	0.212^***^	0.289^***^	0.203^***^								
Friends at work (5)	0.182^***^	0.240^***^	0.169^***^	0.307^***^							
Depressed at work (6)	–0.165^***^	–0.189^***^	–0.076^*^	–0.047	–0.128^***^						
Life satisfaction (7)	0.384^***^	0.202^***^	0.204^***^	0.224^***^	0.106^**^	–0.207^***^					
Happiness in life (8)	0.293^***^	0.207^***^	0.169^***^	0.203^***^	0.164^***^	–0.182^***^	0.403^***^				
Meaning in life (9)	0.302^***^	0.194^***^	0.231^***^	0.219^***^	0.145^***^	–0.235^***^	0.531^***^	0.486^***^			
Purpose in life (10)	0.270^***^	0.190^***^	0.184^***^	0.168^***^	0.136^***^	–0.145^***^	–0.289^***^	0.386^***^	0.590^***^		
Friends in life (11)	0.303^***^	0.184^***^	0.081^*^	0.185^***^	0.225^***^	–0.119^***^	0.249^***^	0.323^***^	0.293^***^	0.193^***^	
Depressed in life (12)	–0.193^***^	–0.177^***^	–0.097^**^	–0.106^**^	–0.080^*^	0.169^***^	–0.162^***^	–0.234^***^	–0.168^***^	–0.087^**^	–0.155^***^

*** *p < 0.001*,

** *p < 0.01*,

* *p < 0.05*.

## Footnotes

^1^SWB is defined as either a cognitive or affective evaluation of life, and is usually assessed for life as a whole, or for specific facets (e.g., life at work).
